# Health laboratory licensing: a policy and best-practice analysis

**DOI:** 10.2471/BLT.24.292760

**Published:** 2025-05-03

**Authors:** Markus Huber, Madina Andreyeva, Lance D Presser, Joanna Salvi Le Garrec Zwetyenga

**Affiliations:** a Bio-risk Management and Biosafety, WHO Health Emergencies Programme, World Health Organization, Avenue Appia 20, 1211 Geneva, Switzerland.; b Better Labs for Better Health initiative, World Health Organization Regional Office for Europe, Copenhagen, Denmark.; c Centre for Infectious Diseases Research, Diagnostics and Laboratory Surveillance, National Institute for Public Health and Environment, Bilthoven, Kingdom of the Netherlands.

## Abstract

**Objective:**

To gain insight into how governments regulate their health laboratory sector, by reviewing health laboratory licensing legislation across different health-care systems in a diverse range of 18 countries worldwide.

**Method:**

We selected countries for a diverse range of health-care systems, geography, income level, licensing legislation and standards adhered to. We selected aspects of health laboratory licensing that were consistently present in different countries and suitable for meaningful comparison, focusing on legislative approaches, certification and accreditation models, regulation, quality assurance, and biosafety and biosecurity requirements.

**Findings:**

Our analysis revealed that the licensing legislation for health laboratories typically encompasses two principal components: administrative procedural law and substantive law. We observed that the different ways in which countries regulate their health laboratories could be categorized within three distinct legislative approaches, namely: standalone licensing act, general licensing act and one based on a health insurance contract. Most countries used a two-step application process, comprising registration and licensing steps. License validity periods ranged over 1–5 years, with some countries opting for permanent licenses. Countries adopted diverse standards and requirements, with some mandating accreditation.

**Conclusion:**

Our findings highlight the diverse legislative approaches to health laboratory licensing, reflecting varying national capacities and regulatory priorities. Integrating robust quality standards, especially those aligned with International Organization for Standardization standard no. 15189, is essential for strengthening laboratory oversight and public health response. Effective licensing frameworks not only enhance domestic laboratory systems but also contribute to global health security through alignment with international obligations.

## Résumé

**Objectif:**

Comprendre la manière dont les pouvoirs publics réglementent le secteur des laboratoires de santé, en examinant les législations relatives à l’accréditation des laboratoires de santé dans différents systèmes de soins de santé parmi 18 pays dans le monde.

**Méthode:**

Nous avons sélectionné les pays en fonction de la diversité de leurs systèmes de soins de santé, de leur situation géographique, de leur niveau de revenu, de leur législation en matière d’agrément et des normes qu’ils respectent. Nous avons sélectionné les caractéristiques d’agrément des laboratoires de santé qui étaient systématiquement présents dans les différents pays et qui se prêtaient à une comparaison pertinente, en nous concentrant sur les approches législatives, les modèles de certification et d’accréditation, la réglementation, l’assurance qualité et les exigences en matière de biosécurité et de biosûreté.

**Résultats:**

Notre analyse a révélé que les législations relatives à l’agrément des laboratoires de santé comprend généralement deux composantes principales: le droit procédural administratif et le droit matériel. Nous avons observé que les différentes manières dont les pays réglementent leurs laboratoires de santé se classent dans trois approches législatives distinctes, à savoir: la législation sur l’agrément autonome, la législation sur l’agrément général et la législation fondée sur un contrat d’assurance maladie. La plupart des pays appliquent une procédure de demande en deux étapes, comprenant l’enregistrement et l’octroi d’un agrément. La durée de validité des agréments varie de 1 à 5 ans, certains pays optant pour des agréments permanents. Les pays ont adopté des normes et des exigences diverses, certains imposant l’accréditation.

**Conclusion:**

Nos résultats mettent en évidence la diversité des approches législatives en matière d’octroi d’agréments aux laboratoires de santé, qui reflète la diversité des capacités nationales et des priorités réglementaires. L’intégration de normes de qualité robustes, en particulier celles conformes à la norme nº 15189 de l’Organisation internationale de normalisation, est essentielle pour renforcer la surveillance des laboratoires et la réponse en matière de santé publique. Des cadres efficaces d’octroi d’agréments permettent non seulement de renforcer les systèmes nationaux de laboratoires de santé, mais contribuent également à la sécurité sanitaire mondiale en se mettant en conformité avec les obligations internationales.

## Resumen

**Objetivo:**

Analizar cómo los gobiernos regulan el sector de los laboratorios de salud, mediante la revisión de la legislación sobre licenciamiento de laboratorios en diversos sistemas sanitarios de 18 países del mundo con contextos heterogéneos.

**Métodos:**

Se seleccionaron países que representaran una amplia variedad de sistemas sanitarios, regiones geográficas, niveles de ingreso, legislación sobre licencias y estándares adoptados. Se eligieron aspectos del licenciamiento de laboratorios de salud que estuvieran presentes de manera constante en distintos países y fueran aptos para una comparación significativa, con énfasis en los enfoques legislativos, modelos de certificación y acreditación, regulación, aseguramiento de la calidad y requisitos de bioseguridad y bioprotección.

**Resultados:**

El análisis reveló que la legislación sobre licenciamiento de laboratorios de salud suele abarcar dos componentes principales: la normativa procedimental administrativa y la normativa sustantiva. Se observó que los distintos enfoques nacionales para regular los laboratorios de salud pueden agruparse en tres modelos legislativos diferenciados: ley de licencias independiente, ley general de licencias y modelo basado en contrato de seguro de salud. La mayoría de los países emplea un proceso de solicitud en dos etapas, que comprende un registro inicial seguido de la obtención de la licencia. La vigencia de las licencias varía entre 1 y 5 años, aunque algunos países han optado por licencias permanentes. Se constató una gran diversidad de normas y requisitos. Además, en algunos casos se exige la acreditación obligatoria.

**Conclusión:**

Los resultados destacan la diversidad de enfoques legislativos en el licenciamiento de laboratorios de salud, lo que refleja capacidades nacionales distintas y prioridades regulatorias diversas. La integración de estándares de calidad sólidos, en especial aquellos alineados con la norma n.º 15189 de la Organización Internacional de Normalización, resulta fundamental para reforzar la supervisión de los laboratorios y la respuesta en salud pública. Los marcos eficaces de licenciamiento no solo fortalecen los sistemas de laboratorio nacionales, sino que también contribuyen a la seguridad sanitaria mundial mediante la alineación con compromisos internacionales.

## ملخص

الغرض فهم كيفية تنظيم الحكومات لقطاع المختبرات الصحية لديها، من خلال مراجعة تشريعات ترخيص المختبرات الصحية عبر مختلف أنظمة الرعاية الصحية في مجموعة متنوعة من 18 دولة حول العالم.

الطريقة اخترنا دولًا بناءً على تنوع أنظمة الرعاية الصحية، والموقع الجغرافي، ومستوى الدخل، وتشريعات الترخيص، والمعايير التي تلتزم بها هذه الدول. اخترنا جوانب ترخيص المختبرات الصحية التي كانت موجودة باستمرار في مختلف الدول، والتي كانت مناسبة للمقارنة الهادفة، مع التركيز على الأساليب التشريعية، ونماذج التصديق والاعتماد، والتنظيم، وضمان الجودة، ومتطلبات السلامة والأمن البيولوجي.

النتائج كشف التحليل أن تشريعات ترخيص المختبرات الصحية تشمل عادةً عنصرين رئيسيين: قانون الإجراءات الإدارية، والقانون الموضوعي. ولاحظنا أن الطرق المختلفة التي تنظم بها الدول مختبراتها الصحية يمكن تصنيفها ضمن ثلاثة أساليب تشريعية متميزة، وهي: قانون الترخيص المستقل، وقانون الترخيص العام، وقانون الترخيص القائم على عقد التأمين الصحي. اعتمدت معظم الدول عملية تقديم طلبات من خطوتين، تتضمن التسجيل والترخيص. تراوحت فترات صلاحية التراخيص بين 1 و5 سنوات، واختارت بعض الدول التراخيص الدائمة. وتبنت الدول معايير قياسية ومتطلبات متنوعة، مع إلزام بعضها بالاعتماد.

الاستنتاج تلق نتائجنا الضوء على تنوع الأساليب التشريعية المتبعة في ترخيص المختبرات الصحية، مما يعكس تباين القدرات الوطنية والأولويات التنظيمية. إن دمج معايير جودة متينة، وخاصةً تلك المتوافقة مع معيار المنظمة الدولية للمعايير رقم 15189، يُعدّ أمرًا ضروريًا لتعزيز الرقابة على المختبرات واستجابة الصحة العامة. ولا تقتصر أطر عمل الترخيص الفعالة على تعزيز أنظمة المختبرات المحلية فحسب، بل تُسهم أيضًا في الأمن الصحي العالمي من خلال تنسيقها مع الالتزامات الدولية.

## 摘要

**目的:**

通过研究全球 18 个国家的不同卫生保健系统中的保健实验室许可制立法情况来深入了解各国政府是如何监管其保健实验室部门的。

**方法:**

我们根据多样化卫生保健系统、地理环境、收入水平、许可制立法情况以及所遵循标准对各个国家进行了筛选。我们选取了不同国家的保健实验室许可制中始终存在且适用于进行有意义比对的各个要素，并把焦点集中于立法途径、审定及认证模式、法规、质量保证以及生物安全和生物安保要求。

**结果:**

根据我们的分析，保健实验室许可制立法通常包括两个主要组成部分，分别是行政程序法和实体法。据我们观察，各国对其保健实验室采取的不同监管方式可被归类为三种不同的立法途径，即独立许可法、通用许可法以及基于健康保险合同的立法。大多数国家采用两步申请流程，该流程包括登记和许可两个步骤。许可证有效期为 1 至 5 年不等，但有些国家选择颁发永久许可证。各国采用不同的标准和要求，其中有些国家强制要求认证。

**结论:**

我们的研究结果突出了各国保健实验室许可制的多样化立法途径，这反映了各国在能力和监管优先事项方面的差异。将稳健的质量标准（尤其是与国际标准化组织第 15189 号标准相契合的此类标准）进行整合，这是加大实验室监督力度和提高公共卫生应对能力的必行之举。有效的许可制框架不仅可以增强本国实验室系统，还可通过与国际义务相契合来促进全球卫生安全。

## Резюме

**Цель:**

Изучить механизмы государственного регулирования сектора медицинских лабораторий путем анализа законодательства о лицензировании лабораторий в различных системах здравоохранения. В качестве примера были взяты 18 стран, представляющих различные подходы к организации здравоохранения.

**Методы:**

Были отобраны страны с самыми различными типами систем здравоохранения, отличающиеся по географическому положению, уровню доходов населения, характеру законодательства о лицензировании и применимым стандартам. Авторы выделили аспекты лицензирования медицинских лабораторий, которые в равной мере присутствовали во всех странах и допускали содержательное сравнение, уделяя особое внимание законодательным подходам, моделям сертификации и аккредитации, нормативным актам, обеспечению качества и требованиям по части биологической защиты и биологической безопасности.

**Результаты:**

Анализ показал, что законодательство о лицензировании медицинских лабораторий обычно включает два основных компонента: административно-процессуальное законодательство и материально-правовые нормы. Авторы отмечают, что отличия в регулировании медицинских лабораторий в различных странах могут быть сведены к трем четко различающимся подходам, а именно: отдельному специализированному закону о лицензировании, общему закону о лицензировании и контракту о медицинском страховании. Большинство стран используют двухступенчатый процесс подачи заявки, включающий этапы регистрации и лицензирования. Срок действия лицензии составляет от 1 года до 5 лет, но некоторые страны предлагают возможность получения бессрочной лицензии. Страны используют разные стандарты и требования, некоторые из них требуют аккредитации.

**Вывод:**

В заключении подчеркивается, что в мире наблюдается значительное разнообразие подходов к законодательному регулированию лицензирования медицинских лабораторий, связанное с различиями в национальных возможностях и законодательных приоритетах. Внедрение жестких международных стандартов качества, особенно гармонизированных со стандартом Международной организации по стандартизации № 15189, крайне важно для укрепления надзора над лабораториями и для реагирования в сфере общественного здравоохранения. Внедрение эффективных парадигм лицензирования не только укрепляет местные лабораторные системы, но и вносит вклад в безопасность здравоохранения в мировом масштабе за счет согласования с международными обязательствами.

## Introduction

Health laboratories, including public health laboratories, private and public clinical laboratories, and national reference laboratories, are integral to public health systems, performing activities such as the detection, assessment, notification and monitoring of acute health events, as described in annex 1 of the International Health Regulations (IHR) (2005).^1^ The monitoring and evaluation of laboratory capacity to perform such activities is critical to ensure compliance with these regulations.^2^

A health laboratory license is the formal authorization granted by a regulatory body permitting a laboratory to conduct specific activities in accordance with established legal and quality standards and requirements. The issuance of a license, in either physical or digital form, establishes the foundation for the operation and management of a laboratory. The license serves as a key regulatory mechanism to ensure laboratories operate safely, maintain high-quality testing practices, and comply with public health and safety requirements. However, the World Health Organization (WHO) Better Labs for Better Health initiative has identified significant deficiencies (e.g. inconsistent licensing requirements, insufficient oversight mechanisms and a lack of enforcement) in the national regulatory frameworks of health laboratories in some Member States of the WHO European Region.^3^^,^^4^ Inadequate regulation poses risks, such as compromised laboratory standards, inaccurate test results and potential harm to patients and the public. These deficiencies highlight the critical role of robust licensing legislation in ensuring the quality and safety of medical testing and diagnostic services.

To gain insight into how governments regulate their health laboratory sector, we reviewed health laboratory licensing legislation in a diverse range of 18 countries across the globe (Armenia, Australia, Austria, Canada, France, Germany, Ghana, Kyrgyzstan, Luxembourg, Malaysia, Malta, Philippines, Russian Federation, Switzerland, Ukraine, United Arab Emirates, United Kingdom of Great Britain and Northern Ireland, and United States of America). Our study aims to support national health authorities and laboratory managers in strengthening their laboratory systems by establishing adequate licensing legislation, ultimately improving the quality of diagnostic services.

## Methods

Our selection of the 18 countries included in this study was guided by key criteria to ensure a comprehensive and representative analysis of health laboratory licensing legislation. These criteria included diversity in health-care systems (public, private and mixed), geographic and economic representation (low-, middle- and high-income countries) and regulatory models (in terms of the different approaches to licensing legislation). We also selected countries for a range of certification and accreditation standards requirements (e.g. International Organization for Standardization standard, ISO 15189^5^ or national alternatives), legislative transparency (publicly accessible or translatable laws) and policy relevance, including both established frameworks and recent reforms.

Three authors collected legislative data independently, each assigned a subset of countries based on their language proficiency. WHO translators translated legislation into English where necessary. Following the initial data collection in January 2024, two authors conducted the analysis, identifying and categorizing the legislative approaches and licensing aspects. The review process compiled overlapping contributions from several authors, with consensus used to address and resolve any inconsistencies.

Because the key aspects of the licensing legislation were not predefined but emerged inductively during our review of national legislation, we did not use a standardized data extraction template. We selected aspects of health laboratory regulation that appeared consistently in different countries and could be meaningfully compared, enabling an analysis of legal requirements, major legislative approaches, certification and accreditation models, regulation, quality assurance, and biosafety and biosecurity requirements. To organize our qualitative data, we used the consolidated criteria for reporting qualitative research framework.^6^

## Results

### Legislative approaches 

Our analysis revealed that the licensing legislation for health laboratories typically encompasses two principal components: administrative procedural law and substantive law. Administrative procedural law is pivotal in delineating the process by which licenses are issued or denied, (i) detailing the precise steps, requirements and criteria to which applicants must adhere; and (ii) providing directives for the licensing authority on how to review and evaluate applications, ensuring a transparent and standardized evaluation process. Substantive law either (i) specifies and delineates all standards and requirements of the licensing framework; or (ii) refers to quality, labour or safety regulations, or to established guidelines. We observed that the different ways in which these separate components are enacted could be categorized within three distinct legislative approaches, namely: a standalone licensing act, a general licensing act or a model based on a health insurance contract. Table 1 presents the identified properties of each approach. 

**Table 1:** Properties of three main legislative approaches in a multi-country comparison of health laboratory legislation

Property	Standalone licensing act	General licensing act	Based on health insurance contract
Character of the legal acts	A single specific legal act	A single general legal act on licensing and more specific legal acts relating to the operation of the laboratory	A single act regulating the contract between the laboratory and the insurance company, and more specific legal acts
Quality enforcement	Controlling authority	Controlling authority	Controlling authority and insurance companies
Governmental supervision	Significant	Significant	Moderate
Application process	Registration and/or licensing	Registration and/or licensing	Personnel registration and contract with insurer
License function	Permission to operate	Permission to operate	Basis for reimbursement

#### Standalone licensing act

This approach concentrates most regulatory aspects that govern health laboratories into a single legislative act, combining administrative procedural law and substantive law. The act covers the application process and the required documentation to be submitted to the licensing authority, and is often accompanied by a list of minimum criteria to ensure the quality and safety of the operations of the health laboratory. Through the act, the government regulates the reasons for granting, refusing or renewing licenses; license validity period; the implementation of licensing control; and the suspension, renewal, termination and revocation of licenses. We noted that health laboratory licensing follows this approach in Canada (specifically, Saskatchewan), France, Malaysia, Malta and the Philippines (Table 2). 

**Table 2:** Health laboratory license and application process comparison for 18 countries, 2024

Country	Legislative approach	Character of awarded license	License validity period	Application process	Application fees
Armenia	General	License to operate a medical laboratory	5 years	Single-step process: applying for a license with licensing agency, health ministry	License fee set by law on state fees
Australia	Health insurance	Accreditation to operate a medical laboratory	3 years	Two-step process: laboratories obtain accreditation from the National Association of Testing Authorities; laboratory scientists register with the Australian Health Practitioner Regulation Agency	Fees vary depending on the accreditation and registration processes
Austria	Health insurance	License to operate a medical laboratory	Permanent	Two-step process: registration as the laboratory manager; signing a contract with the insurance company	Federal medical board registration fee
Canada	Standalone	License to operate a medical laboratory	1 year	Two-step process: application for a license; mandatory participation in quality assurance programme	Fees determined by the health ministry
France	Standalone	Administrative authorization	Permanent	Two-step process: administrative authorization; accreditation process	Costs related to the accreditation process
Germany	Health insurance	Contract with the insurance company	Permanent	Two-step process: registering the laboratory manager; signing a contract with the insurance company	Federal medical board registration fee
Ghana	General	Preliminary license, long-term license	6 months or 3 years	Single-step process: obtain a license from the medicines and medical devices department under the health ministry	License fee and annual retention fee
Kyrgyzstan	General	License to operate a medical laboratory	Permanent	Single-step process: applying with the health authority	Fees determined by the health ministry (may vary according to services offered)
Luxembourg	Health insurance	Contract with the insurance company	Permanent	Two-step process: registering the laboratory manager; contract with the insurance company	Fee set by national authorities
Malaysia	Standalone	Approval and license	1–3 years	Two-step process: application for approval; application for a license within 3 years	Fee set by national authorities
Malta	Standalone	License for private medical diagnostics laboratories	1 year	Two-step process: application submitted to the health-care standards directorate; inspection by the health-care standards directorate	Annual license fee
Philippines	Standalone	License for clinical laboratories	1 year	Two-step process: registering with the health department or Facilities and Services Regulatory Bureau; license application	License fee set by the health department
Russian Federation	General	License to operate a medical laboratory	5 years	Single-step process: registration with health authority and compliance with federal norms	Set by federal authorities
Switzerland	Health insurance	Contract with the insurance company	Permanent or 5 years for microbiological laboratories	Two-step process: registering the laboratory manager; contract with the insurance company	Cost of registering the laboratory manager
Ukraine	General	Preliminary license, accreditation	Permanent	Two-step process: health ministry license; accreditation via national authority	License and accreditation fees
United Arab Emirates	General	Preliminary license, accreditation	1-year preliminary license	Two-step process: online registration and application on the health department’s website; onsite inspection	Fee set by the health department
United Kingdom	Health insurance	Contract with the insurance company	Permanent	Two-step process: registering the laboratory manager; contract with the insurance company	Cost of clinical pathology accreditation
United States	General	Clinical Laboratory Improvement Amendments certificate	Varies by certificate type	Two-step process: certification through Centers for Medicare and Medicaid Services; accreditation by approved bodies	Costs of clinical laboratory improvement amendments

#### General licensing act

This approach establishes a hierarchy of different laws. The administrative procedural law and the substantive law are separated and enforced through different acts. The highest level represents legislation on the general aspects of licensing, which is complemented by legislation specifically addressing licensing of medical and/or public health activities and legislation regarding the substantive law of the laboratory operation. The general licensing act determines the rules for issuance, renewal and withdrawal of any license. A related act describes the requirements for licenses for medical and/or public health activities, mostly accompanied by a document determining the required standards. In contrast to the standalone licensing act, we noted that this approach utilizes general guidelines for any medical and/or public health activity or health facility. We observed that health laboratories in Armenia, Ghana, Kyrgyzstan, the Russian Federation, Ukraine, the United Arab Emirates and the USA followed this legislative approach (Table 2).

#### Based on health insurance contract 

In the third approach, the related acts regulate the contract between the health laboratory service provider and the insurance companies (which are often publicly owned), and establish mandatory requirements to operate the laboratory. Because of significant issues with reimbursement of services provided by health laboratories in the last century (e.g. in the USA), insurance companies established rules and guidelines to ensure quality enforcement and conduct, and participation in external audits.^7^ Referring to these rules and guidelines is often an obligation of health insurance legislation. The health insurance companies use standard contract forms describing the requirements of the laboratory, which can be stricter than those stipulated in the respective legislation. To receive reimbursement, the laboratory service provider must comply with the terms of the contract. In some countries (e.g. Australia, Germany, Luxembourg and the United Kingdom), mandatory participation in a certification or an accreditation programme might be required by such a contract. Our analysis revealed that health laboratories in Australia, Austria, Germany, Luxemburg, Switzerland and the United Kingdom followed a licensing approach based on health insurance contract (Table 2).

### Application process 

We observed that the license application process commonly included two steps: (i) registration (sometimes called preliminary licensing, authorization or approval process); and (ii) licensure. Key aspects of this process are provided in Table 2 for all countries analysed.

The first step entails registering with the national health ministry or any designated authority (e.g. licensing authority). This process is sometimes accompanied by a preliminary approval (e.g. France, Malaysia and United Arab Emirates), during which the licensing authority collects information about the laboratory and ensures compliance with the relevant regulations. The initial registration and review of the legal requirements for the health laboratory can have two different implications. First, they provide a legal basis for the laboratory to establish its service (the registration alone does not authorize the provision of services to clients). Second, it provides the legal basis to establish the laboratory and authorizes the laboratory owner to provide services to clients. This option is used when accreditation or certification is mandatory for obtaining a license (e.g. Australia, France, Ukraine, United Kingdom and USA). Since accreditation and certification can only be properly evaluated in daily operations, the laboratory has to provide services to clients before the license application. The legal requirements for registration in this scenario are stricter and often accompanied by an initial onsite inspection, and the laboratory must apply for its registration to the licensing authority before the effective start of operations.

The second step is the licensure process of the laboratory, during which the licensing authority verifies the information collected during the registration process and gathers new information from the laboratory, preferably from daily operations. The laboratory applies for the license after completing the registration with the licensing authority. 

In some countries (e.g. Armenia, Ghana, Kyrgyzstan and Russian Federation), the application process is limited to a single step, and registration is conducted when the application for a license is being processed by the licensing authority.

### Validity period

We noted that licenses are usually issued for a period of 1–3 years, so that licensing authorities can regularly determine whether laboratories are continuing to fulfil their licensing requirements. The health laboratory licensing legislation usually outlines the process for license renewal. We noted that permanent licenses are issued in France, Germany, Kyrgyzstan, Luxemburg, Ukraine and the United Kingdom, but these permanent licenses can be temporarily suspended or revoked if the health laboratory fails to meet licensing requirements. We provide license validity periods for all countries analysed in Table 2.

### Fees

We observed that all countries prescribe a registration and/or a licensing fee (Table 2). In some countries (e.g. Malta, Philippines, Ukraine and United Arab Emirates) these fees cover the cost of onsite inspection for the registration and licensing process, as well as the cost of the entire administrative process.

### Standards and requirements

We noted that health laboratories in the analysed countries regulate the legal standards and requirements concerning the structure, functions, personnel requirements and other related aspects either in their licensing legislation or by referring to applicable laws or guidelines. For example, in the Philippines, Switzerland and the United Arab Emirates, the laboratory licensing laws are accompanied by a publicly available compilation of all applicable laws and regulations for laboratories.

We also observed that the practice of implementing standards in licensing legislation varies between countries, with some overlap (Table 3). The relevant authorities publish and update a minimum criteria list in 12 of the 18 countries analysed (Armenia, Australia, Canada, Ghana, Kyrgyzstan, Luxemburg, Malta, Philippines, Russian Federation, Ukraine, United Kingdom and USA); developed an individual country-specific mandatory accreditation in six countries (Australia, Canada, France, Luxembourg, United Kingdom and the USA); regulate only certain aspects of all possible requirements and standards observed in their licensing legislation in two countries (Austria and Malaysia); and chose mandatory ISO 15189 accreditation as a requirement for the laboratory service provider either to operate or to obtain reimbursement from the insurance companies in one country (France). Health laboratories in seven countries (Australia, France, Germany, Luxembourg, Switzerland, United Arab Emirates and United Kingdom) are regulated by the relevant authorities with standards and requirements that are based on ISO 15189.

**Table 3:** Source of health laboratory licensing standards and requirements for the 18 countries analysed, 2024

Country	Main list or source of legal acts for standards and requirements	Implementation	Year of enactment
Armenia	Governmental Decree of Republic of Armenia no. 867, 29 June 2002^8^^,a^	Minimum criteria list^b^	2002
Australia	Requirements for medical pathology services, third edition, 2018^9^	Mandatory accreditation based on a minimum criteria list (based on ISO 15189)^c^	2018
Austria	Guidelines to the laboratory-catalogue, Hauptverband der Sozialversicherungen, 2018^10^	Requirements have to be met	2018
Canada	The Medical Laboratory Licensing Regulations, 2009^11^	Mandatory accreditation based on a minimum criteria list	1995
France	Order no. 2010–49 relative to the medical biology, 13 January 2010^12^	Mandatory ISO 15189 accreditation	2010
Germany	Guidelines of the Bundesärztekammer for quality assurance: medical laboratory examinations, Richtlinie der Bundesärztekammer zur Qualitätssicherung laboratoriumsmedizinischer Untersuchungen (Rili-BÄK), 2019^13^	Guideline based on ISO 15189	2019
Ghana	National guidelines for laboratory testing and reporting on respiratory infectious diseases in health facilities in Ghana, first edition, 2020^14^	Minimum criteria list	2011
Kyrgyzstan	Decree of the Kyrgyz Republic Cabinet of Ministers no. 678, 14 December 2023^15^^,a^	Minimum criteria list	2024
Luxembourg	Grand-Ducal regulation of 27 May 2004 determining the minimum criteria for medical analysis laboratories^16^	Minimum criteria list or mandatory ISO 15189 accreditation	2004
Malaysia	Act 674 Pathology laboratory act, 2007^17^	Requirements have to be met	2007
Malta	Ministry for Health and Active Ageing, Healthcare Standards Directorate, Superintendence of Public Health 2019^18^	Minimum criteria list	2019
Philippines	Assessment tool for licensing a general clinical laboratory, 2021^19^	Minimum criteria list	2021
Russian Federation	Governmental Decree of the Russian Federation no. 852, 1 June 2021^20^	Minimum criteria list	2021
Switzerland	Kriterien zum Betreiben von medizinischen Laboratorien (KBMAL) 3.0, 2017^21^	Guideline based on ISO 15189	2017
Ukraine	Cabinet of Ministers of Ukraine Decree no. 285, 2 March 2016^22^	Minimum criteria list	2016
United Arab Emirates	Health Authority Abu Dhabi clinical laboratory standards, 2011^23^	Mandatory ISO 15189 accreditation	2011
United Kingdom	Care Quality Commission Guidance for providers on meeting the regulations, 2015^24^	Clinical Pathology Accreditation;^d^ minimum criteria list (based on ISO 15189)	2015
United States	Title 42 – The public health and welfare act, Subpart 2 – Clinical laboratories^25^	Clinical Laboratory Improvement Amendments;^e^ minimum criteria list or College of American Pathologists accreditation^f^	1988

ISO: International Organization for Standardization.

^a^ A regulation relating to the standards and requirements was at draft stage at the time of analysis.

^b^ A comprehensive list of standards and requirements is incorporated in the respective legislation.

^c^ ISO 15189: 2022 medical laboratory accreditation: requirements for quality and competence.

^d^ Clinical Pathology Accreditation (United Kingdom) Ltd provides a means to accredit clinical pathology services and external quality assessment schemes.^26^

^e^ A series of amendments (Title 42 Code of Federal Regulations Part 493: Clinical Laboratory Improvement Amendments) for establishing laboratory standards.^27^

^f^ The College of American Pathologists accredits laboratories testing specimens from humans or animals using methodologies and clinical application within the expertise of the programme. Laboratories must be appropriately licensed to perform testing when required by law. College of American Pathologists accreditation meets the required Clinical Laboratory Improvement Amendments standards.

The sources of law for each country are provided in the online repository^28^ and in Table 3.

### Quality management content

**Fig. 1:**
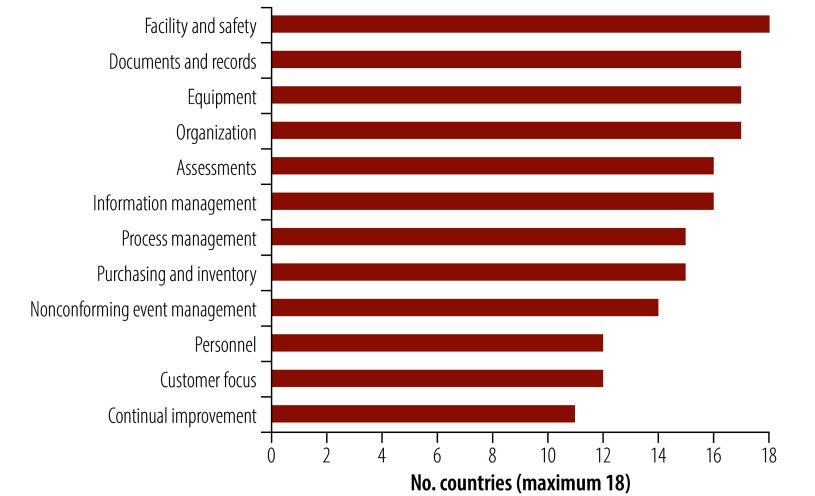
Number of countries including each quality system essential in their health laboratory licensing process requirements, 18 countries, 2024

We compared the quality management content of the various existing legislations for laboratory licensing with the 12 quality system essentials defined by the Clinical and Laboratory Standards Institute.^29^ Fig. 1 indicates the number of countries out of the 18 analysed in which each of the quality system essentials was included in the requirements of the health laboratory licensing legislation. The analysis does not provide any assessment of the enforcement mechanisms or qualitative robustness of each component in the licensing legislation. Our data reveal that although core operational quality system essentials are widely adopted in health laboratory licensing, more strategic and progressive quality elements, such as customer focus and continual improvement, remain underrepresented. 

## Discussion

Our comparative review of health laboratory licensing legislation in 18 countries has highlighted the significant diversity in regulatory approaches and the absence of a universally adopted framework, but also a common goal of safeguarding public health through effective laboratory oversight. Our findings reveal broad alignment on infrastructure and operational domains, but limited regulatory attention to continuous quality improvement, highlighting the need for more comprehensive quality assurance integration globally.

We identified three major legislative approaches, each of which presents unique mechanisms for oversight and quality control, affecting laboratory performance differently. Understanding the relative strengths and limitations of these approaches will help policy-makers to choose and adapt frameworks that can sustainably enhance laboratory quality and ultimately protect public health. The standalone licensing approach offers the most comprehensive form of oversight, as it is tailored specifically to laboratory operations. Countries that implement this model typically demonstrate strong alignment with international standards and more robust mechanisms for inspection, enforcement and continuous quality improvement. These frameworks tend to have a direct, positive impact on laboratory performance by ensuring that only facilities meeting defined standards are allowed to operate. The general licensing approach, in which laboratory licensing is embedded within broader health-care or institutional frameworks, may provide less specialized attention to laboratory-specific requirements. Although more administratively efficient, this model may struggle to ensure consistent performance across laboratory types unless paired with strong, sector-specific guidelines. Licensing approaches based on health insurance contracts regulate laboratories through contractual obligations tied to reimbursement by insurance providers. Although this model can incentivize compliance with performance standards, especially in privatized health systems, it may lack the regulatory authority to enforce sanctions or mandate corrective actions in the public interest.

The effectiveness of any licensing model is context dependent. For example, in countries with a strong public health infrastructure and sufficient regulatory capacity, a standalone licensing act can be effectively implemented and enforced. Conversely, in settings with constrained resources or decentralized governance, general or contract-based models may be more feasible initially, even if less robust. Cultural, legal and economic contexts also shape the success of licensing models. In highly privatized health systems, models based on health insurance contracts may leverage market mechanisms to drive compliance. What the three approaches have in common is that they impact not only the legal instruments used and standards and requirements enforced, but also the responsibilities of regulatory authorities and insurers, especially in systems where insurance providers play a central role in enforcing quality standards.

Another critical dimension of licensing legislation is the enforcement of standards and quality management systems. Although 12 countries maintain a published list of minimum criteria, and others incorporate or require accreditation (often aligned with ISO 15189), the depth and breadth of quality assurance integration vary widely. Moreover, the role of international standards, particularly ISO 15189, emerges as a potential unifying benchmark. Countries leveraging these standards, whether through direct legal requirements or insurance contracts, may be better positioned to align with global health security objectives and cross-border recognition of laboratory competence. However, without mechanisms to monitor implementation, enforce compliance and evaluate performance, well-drafted legislation may fail to achieve its intended impact.

Our findings are directly relevant to the obligations of countries under IHR (2005).^1^ By establishing clear standards, oversight processes and mechanisms for accountability, licensing systems support the functional capacity of national laboratories and their integration into public health surveillance networks. Licensing mechanisms help establish minimum standards for infrastructure, staff qualifications, equipment, procedures, quality control and biosafety, which are essential for delivering accurate and timely diagnostic services. By enforcing these standards, licensing promotes consistency, accountability and alignment with international best practice.

Our study had several limitations in terms of scope. First, the laboratory sector is subject to a comprehensive framework of laws and regulations, extending beyond the remit of this review. Within a single country, various laws and regulations may govern different types of health laboratories, leading to potential variations in the licensing process, application procedures, and standards and requirements for compliance. Although we made efforts to accurately categorize countries according to specific licensing approaches, different laboratories within a single country may exhibit diverse licensing processes. Second, our study did not include consultations with national authorities or stakeholders, which would be a valuable component for future research to validate and contextualize findings. Finally, countries without formal laboratory licensing systems were not assessed, as the study focused on analysing and comparing existing legislative frameworks; our findings may therefore not be globally generalizable.

To conclude, our findings suggest that effective licensing frameworks must go beyond administrative compliance. Licensing should function as a strategic instrument to strengthen laboratory systems, enabling countries to implement robust quality management systems; ensure adherence to biosafety and biosecurity protocols; improve accountability and transparency; and facilitate data sharing and public health response coordination. Supporting efforts to harmonize standards and requirements, particularly in regions with frequent cross-border health challenges, could significantly strengthen domestic health systems but also advance global health security and collective preparedness.
